# GLP-1 Receptor Agonists or Dual GLP-1/GIP Receptor Agonists vs. SGLT2 Inhibitors in Patients with Atrial Fibrillation and HFpEF: A Propensity-Matched Real-World Analysis

**DOI:** 10.3390/jcm15134992

**Published:** 2026-06-26

**Authors:** Faizan Ahmed, Najam Gohar, Madeeha Shafqat, Daniel Aziz, Mohammad Omar Butt, Hassaan Abid, Haziq Ahmad, Mohammad Saad Saeeduddin, Ch M Umer Zaman, Haris Bin Tahir, Muhammad Hassan, Qaiser Shahzad, Ayesha Zulfiqar, Amro Taha, Swapnil Patel, Eran S. Zacks

**Affiliations:** 1 Department of Internal Medicine, Hackensack Meridian Health, Jersey Shore University Medical Center, Neptune, NJ 07753, USA; swapnil.patel@hmhn.org; 2 Department of Medicine, Ameer-ud-Din Medical College, Lahore 54000, Pakistan; najamgoharr@gmail.com (N.G.); omarzaman1000@gmail.com (C.M.U.Z.); 3 Department of Internal Medicine, Geisinger Medical Center, Danville, PA 17822, USA; drmadeehashafqat@gmail.com; 4 Division of Cardiology, Gagnon Cardiovascular Institute, Morristown Medical Center, Morristown, NJ 07960, USA; daniel.aziz2@atlantichealth.org; 5 Department of Internal Medicine, Memorial Satilla Health, Waycross, GA 31501, USA; 6 Department of Internal Medicine, Indiana University School of Medicine, Muncie, IN 46202, USA; habid@iu.edu; 7 Department of Internal Medicine, Shaikh Khalifa Bin Zayed Al Nahyan Medical and Dental College, Lahore 54600, Pakistan; haziqahmadmbbs@gmail.com; 8 Department of Internal Medicine, St. Mary Medical Center, Langhorne, PA 19047, USA; mssaeeduddin@gmail.com; 9 Department of Internal Medicine, Lahore General Hospital, Lahore 54000, Pakistan; harristahirchh@gmail.com; 10 Department of Internal Medicine, Dow University of Health Sciences, Karachi 74200, Pakistan; azamh5223@gmail.com (M.H.); ayeshazulfiqar1725@gmail.com (A.Z.); 11 Department of Internal Medicine, Winchester Medical Center, Winchester, VA 22601, USA; qshahzad@valleyhealthlink.com; 12 Division of Cardiology, West Virginia University, Morgantown, WV 26506, USA; taha61591@gmail.com; 13 Department of Electrophysiology, Hackensack Meridian Health, Jersey Shore University Medical Center, Neptune, NJ 07753, USA; erzacks@drzacks.com

**Keywords:** atrial fibrillation, HFpEF, GLP-1 receptor agonists, GLP-1/GIP receptor agonists, SGLT2 inhibitors, cardiovascular outcomes, incretin-based therapy

## Abstract

**Background**: Atrial fibrillation (AF) and heart failure with preserved ejection fraction (HFpEF) usually coexist and are related to increased morbidity and mortality. Cardiovascular benefits have been demonstrated by drugs such as sodium-glucose cotransporter-2 inhibitors (SGLT2i) and GLP-1 receptor agonists including the dual GIP/GLP-1 receptor agonist tirzepatide (collectively, incretin-based therapies); however, their relative effectiveness in patients with concomitant AF and HFpEF remains undefined. **Methods**: We conducted a retrospective, propensity score-matched cohort study utilizing the TriNetX Global Collaborative Network. Adults with AF or atrial flutter with a diagnosis of HFpEF who initiated incretin-based therapies (GLP-1 receptor agonists or dual GLP-1/GIP receptor agonists) or SGLT2i were included; index medication was required to be initiated within 30 days of a qualifying AF/HFpEF diagnosis. 1:1 matching was performed based on baseline medications, demographics, and comorbidities. Co-primary outcomes were all-cause mortality, inpatient visits, and emergency department (ED) visits at 1 year. Secondary outcomes included myocardial infarction, ischemic stroke, acute kidney injury, transient ischemic attack, major adverse cardiovascular events (MACE; all-cause mortality/MI/stroke composite), and AF-related procedures. Agent-specific subgroup analyses were performed for semaglutide and tirzepatide separately. Sensitivity analyses were conducted at 6 months and 2 years. **Results**: 7624 patients were included in each cohort after matching (mean age: 70.8 years; 52% women). At 1 year, incretin-based therapy was associated with lower all-cause mortality (5.3% vs. 7.3%, HR 0.721, 95% CI 0.634–0.820; *p* < 0.001), fewer inpatient visits (30.0% vs. 37.4%, HR 0.743, 95% CI 0.702–0.787; *p* < 0.001), and no statistically significant difference in ED visits (27.0% vs. 28.0%; HR 0.946, 95% CI 0.888–1.007; *p* = 0.081) compared with SGLT2i. Incretin-based therapy was also associated with lower risk of MACE (HR 0.709), acute kidney injury (HR 0.751), myocardial infarction (HR 0.583), catheter ablation (HR 0.685), and electrical cardioversion (HR 0.472). No significant differences were observed in ischemic stroke or transient ischemic attack. These findings were broadly consistent at 6-month and 2-year follow-up, and directionally consistent in agent-specific subgroup analyses of semaglutide and tirzepatide. **Conclusions**: In this large propensity-matched cohort of patients with AF and HFpEF, initiation of incretin-based therapy (GLP-1 receptor agonists or dual GLP-1/GIP receptor agonists) was associated with lower all-cause mortality, fewer inpatient visits, and reduced cardiovascular events compared with SGLT2i. These findings, while subject to observational limitations, suggest potential benefits of incretin-based therapy in this high-risk population and support the need for prospective comparative trials.

## 1. Introduction

Atrial fibrillation (AF) and heart failure with preserved ejection fraction (HFpEF) are two highly prevalent cardiovascular conditions that frequently coexist and synergistically worsen prognosis. AF affects millions of adults worldwide and is associated with increased risks of stroke, hospitalization, and mortality, particularly when present with heart failure syndromes. HFpEF accounts for roughly half of all heart failure cases and continues to rise with the aging population and growing burden of cardiometabolic comorbidities such as obesity and diabetes, yet effective therapies to improve survival remain limited [1,2].

Sodium-glucose cotransporter-2 inhibitors (SGLT2i), initially developed for type 2 diabetes, have emerged as a cornerstone of HFpEF management, demonstrating reductions in heart failure hospitalizations and cardiovascular events irrespective of glycemic status [3]. Beyond their metabolic actions, SGLT2i exert pleiotropic cardioprotective effects such as modulation of cardiac energetics, reduction in inflammation, and attenuation of myocardial stress, which may also mitigate atrial remodeling and incident AF [4]. Data suggest that SGLT2i use is associated with lower rates of new-onset AF and reduced heart failure-related outcomes compared with other antihyperglycemic strategies [5,6].

Glucagon-like peptide-1 receptor agonists (GLP-1RAs), including both selective GLP-1 receptor agonists (e.g., liraglutide, semaglutide, dulaglutide) and the dual GIP/GLP-1 receptor agonist tirzepatide, have similarly demonstrated robust cardiovascular benefits, including reductions in major adverse cardiovascular events and improvements in heart failure outcomes in select populations, particularly those with obesity [7]. Mechanistically, GLP-1RAs promote weight loss, improve endothelial function, reduce systemic inflammation, and ameliorate metabolic dysfunction, factors that are pathophysiologically relevant in HFpEF and possibly AF [8,9]. Recent HFpEF-focused trials highlight potential symptomatic and clinical benefits of GLP-1RA therapy, though data directly comparing GLP-1RAs with SGLT2i are sparse [9,10].

Despite the widespread use of both drug classes in cardiometabolic disease, there remains an important knowledge gap regarding their comparative effectiveness in patients with coexisting AF and HFpEF, a high-risk subgroup with limited therapeutic options. This study leverages real-world data from the TriNetX database to compare clinical outcomes among patients with AF and HFpEF treated with GLP-1 receptor agonists and dual GIP/GLP-1 receptor agonists (collectively, incretin-based therapies) versus SGLT2 inhibitors. By evaluating mortality, major adverse cardiovascular events, arrhythmia-related procedures, and healthcare use over short- and long-term follow-up, we aim to inform evidence-based therapeutic decision-making in this complex and understudied intersection of arrhythmia and heart failure care.

## 2. Methods

### 2.1. Data Source and Study Design

We conducted a retrospective, propensity score matched cohort study using the TriNetX Research Network (TriNetX, LLC, Cambridge, MA, USA), a federated analytics platform that provides access to de-identified electronic health record data from participating healthcare organizations. This analysis was performed within the Global Collaborative Network, which included 168 contributing healthcare organizations at the time of the query (2 January 2026). Because the TriNetX platform provides de-identified data consistent with the Health Insurance Portability and Accountability Act (HIPAA) Privacy Rule, this study was considered exempt from institutional review board oversight.

The TriNetX platform maps data to standard vocabularies including ICD-10-CM (diagnoses), CPT/HCPCS (procedures), RxNorm (medications), and LOINC (laboratory tests). We report this study in accordance with the STROBE guidance for observational research.

### 2.2. Study Population and Exposure Definition

Adult patients (age 18 years or older) with diagnoses of atrial fibrillation or flutter (ICD-10-CM: I48) and diastolic (congestive) heart failure as a proxy for heart failure with preserved ejection fraction (HFpEF; ICD-10-CM: I50.3) were eligible (Supplementary Table S1). To enrich for a HFpEF population and reduce confounding, we excluded patients with systolic (congestive) heart failure (I50.2), heart transplant status (Z94.1), presence of a heart assist device (Z95.811), pregnancy and puerperium diagnoses (O00-O9A), end stage renal disease (N18.6), dependence on renal dialysis (Z99.2), and encounters for care involving renal dialysis (Z49). Eligible terms were required to have occurred on or before 1 January 2024 (to enable a complete 2-year follow-up).

Patients were assigned to cohorts based on initiation of either (1) GLP-1 receptor agonists or the dual GIP/GLP-1 receptor agonist (lixisenatide, liraglutide, exenatide, albiglutide, dulaglutide, semaglutide, or tirzepatide; collectively, incretin-based therapies) or (2) sodium-glucose cotransporter 2 inhibitors (empagliflozin, dapagliflozin, canagliflozin, ertugliflozin, or sotagliflozin). The distribution of individual agents within each cohort is provided in Supplementary Table S5. To create mutually exclusive comparator cohorts, the incretin-based therapy cohort excluded any recorded SGLT2 inhibitor exposure and the SGLT2 inhibitor cohort excluded any recorded incretin-based therapy exposure. For both cohorts, the first recorded exposure to the index drug class was required to occur after any documented diagnosis of atrial fibrillation/flutter and HFpEF, and within 30 days of any such qualifying diagnosis. The index date was defined as the first day on which the patient met cohort criteria, and outcomes were assessed beginning 1 day after the index date.

### 2.3. Outcomes and Follow-Up Windows

The co-primary endpoints were (1) all-cause mortality, (2) inpatient visits, and (3) emergency department (ED) visits. Because TriNetX does not permit identification of disease-specific admissions (e.g., heart failure-specific or atrial fibrillation-specific hospitalization), inpatient visits and ED visits in this analysis represent all-cause utilization. These outcomes therefore serve as proxy measures of overall healthcare utilization trends between cohorts and should be interpreted with appropriate caution, not as direct measures of HFpEF- or AF-specific hospitalization burden. Secondary endpoints included myocardial infarction, cerebral infarction (ischemic stroke), transient ischemic attack, electrical cardioversion, catheter ablation, major adverse cardiovascular events (MACE), and acute kidney injury. MACE was defined as a composite of all-cause mortality, myocardial infarction, and cerebral infarction (ischemic stroke); notably, this composite incorporates all-cause rather than cardiovascular-specific mortality, as cause-of-death ascertainment is not available within the TriNetX platform. Each component was additionally assessed as an individual outcome. Each outcome analysis was restricted to patients without a documented history of that specific outcome before the start of the follow-up window. The primary analysis evaluated outcomes from day 1 through day 365 after index (1-year window). Sensitivity analyses evaluated outcomes over 180 days (6-month window) and 730 days (2-year window) after index, using the same outcome definitions (Supplementary Table S3).

### 2.4. Covariates and Propensity Score Matching

Baseline covariates were assessed using the TriNetX built-in cohort characterization, including demographics (age, sex, race, and ethnicity), comorbidities, and commonly used cardiovascular and metabolic medications (Supplementary Table S2). We used TriNetX propensity score matching to balance observed baseline characteristics between cohorts. Propensity scores were estimated using logistic regression and patients were matched 1:1 using nearest-neighbor matching within the platform. Covariate balance was assessed using standardized mean differences (SMDs), with SMD less than 0.10 considered adequate balance.

### 2.5. Statistical Analysis

Continuous variables are summarized as mean ± SD and compared using independent-samples *t*-tests; categorical variables are presented as counts (%) and compared using χ^2^ tests, consistent with TriNetX analytic outputs. Time-to-event analyses were evaluated using Kaplan–Meier curves and Cox proportional hazards models, with results summarized as hazard ratios (HRs) and 95% confidence intervals (CIs). Between-group comparisons used the log-rank test. All tests were two-sided and statistical significance was defined as *p* < 0.05. All analyses were performed entirely within the TriNetX platform.

### 2.6. Agent-Specific Subgroup Analyses

To address biological heterogeneity within the incretin-based therapy cohort, pre-specified agent-specific subgroup analyses were performed by separately comparing patients who received semaglutide or tirzepatide as the index agent against independently propensity score-matched SGLT2 inhibitor comparators. Each subgroup was matched 1:1 using the same covariate set and nearest-neighbor algorithm as the primary analysis. All outcomes were assessed over the same follow-up windows (6 months, 1 year, and 2 years) using identical analytic methods. Results from these analyses are presented in Supplementary Table S6 (semaglutide) and Table S7 (tirzepatide).

## 3. Results

### 3.1. Cohort Selection and Baseline Characteristics

Within the Global Collaborative Network, 8501 patients with atrial fibrillation/flutter and HFpEF met the incretin-based therapy cohort criteria and 26,889 met the SGLT2 inhibitor cohort criteria. After propensity score matching, 7624 patients remained in each matched cohort.

After matching, baseline characteristics were well balanced across cohorts. The matched mean age was 70.8 ± 9.5 years in the incretin-based therapy cohort and 70.8 ± 10.2 years in the SGLT2 inhibitor cohort, and 52.1% and 51.6% were women, respectively. Most patients were White (81.7% vs. 81.6%) and 11.3% vs. 11.2% were Black or African American. Key comorbidities were comparable, including essential hypertension (73.6% vs. 73.0%), type 2 diabetes mellitus (60.3% vs. 62.3%), overweight or obesity (51.6% vs. 51.8%), and chronic kidney disease (31.9% vs. 32.7%). Selected baseline characteristics of the study cohorts before and after propensity score matching are depicted in Table 1, while detailed baseline characteristics are provided in Supplementary Table S4.

### 3.2. One-Year Outcomes (Primary Analysis)

In the 1-year analysis (day 1 through day 365 after index), incretin-based therapy was associated with lower all-cause mortality and fewer inpatient visits compared with SGLT2 inhibitor use. All-cause mortality occurred in 403 of 7593 patients (5.3%) in the incretin-based therapy cohort versus 553 of 7592 patients (7.3%) in the SGLT2 inhibitor cohort (HR 0.721, 95% CI 0.634–0.820; log-rank *p* < 0.001). Inpatient visits occurred in 2127 of 7079 patients (30.0%) in the incretin-based therapy cohort versus 2684 of 7079 patients (37.4%) in the SGLT2 inhibitor cohort (HR 0.743, 95% CI 0.702–0.787; log-rank *p* < 0.001). Emergency department visits occurred in 1909 of 7079 patients (27.0%) versus 1980 of 7079 patients (28.0%), with no statistically significant difference between groups (HR 0.946, 95% CI 0.888–1.007; *p* = 0.081). As noted above, inpatient visits and ED visits represent all-cause utilization rather than disease-specific events and should be interpreted as proxy measures of healthcare utilization trends.

### 3.3. Secondary Outcomes at 1 Year

**Figure 1:**
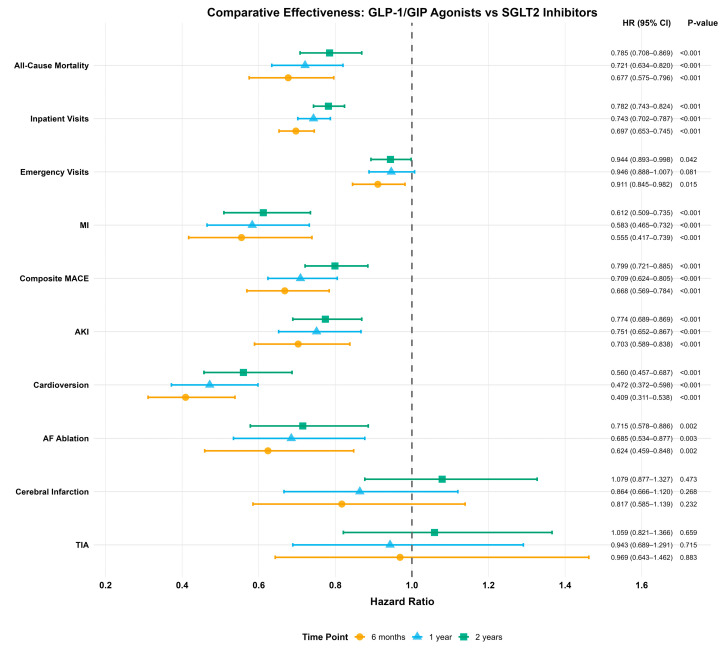
Adjusted HR for primary and secondary outcomes after propensity score matching.

At 1 year, incretin-based therapy was associated with a lower risk of MACE (404/5442 [7.4%] vs. 572/5518 [10.4%]; HR 0.709, 95% CI 0.624–0.805; log-rank *p* < 0.001) and myocardial infarction (118/6312 [1.9%] vs. 202/6359 [3.2%]; HR 0.583, 95% CI 0.465–0.732; log-rank *p* < 0.001). There were no statistically significant differences in cerebral infarction (106/6530 [1.6%] vs. 123/6568 [1.9%]; HR 0.864, 95% CI 0.666–1.120; *p* = 0.268) or transient ischemic attack (76/6962 [1.1%] vs. 80/6955 [1.2%]; HR 0.943, 95% CI 0.689–1.291; *p* = 0.715). Procedural outcomes favored incretin-based therapy, with fewer cardioversions (101/6390 [1.6%] vs. 212/6491 [3.3%]; HR 0.472, 95% CI 0.372–0.598; *p* < 0.001) and catheter ablations (105/6972 [1.5%] vs. 155/7154 [2.2%]; HR 0.685, 95% CI 0.534–0.877; *p* = 0.003). Acute kidney injury was also lower in the incretin-based therapy cohort (330/4732 [7.0%] vs. 440/4800 [9.2%]; HR 0.751, 95% CI 0.652–0.867; *p* < 0.001). After propensity score matching, adjusted HRs for primary and secondary outcomes are shown in Figure 1.

### 3.4. Sensitivity Analyses (6 Months and 2 Years)

Findings were consistent in pre-specified sensitivity analyses as well. At 6 months, incretin-based therapy was associated with lower all-cause mortality (246/7593 [3.2%] vs. 359/7592 [4.7%]; HR 0.677, 95% CI 0.575–0.796; log-rank *p* < 0.001), fewer inpatient visits (1576/7079 [22.3%] vs. 2101/7079 [29.7%]; HR 0.697, 95% CI 0.653–0.745; log-rank *p* < 0.001), and fewer ED visits (1312/7079 [18.5%] vs. 1411/7079 [19.9%]; HR 0.911, 95% CI 0.845–0.982; log-rank *p* = 0.015), along with a lower risk of MACE (251/5442 [4.6%] vs. 376/5518 [6.8%]; HR 0.668, 95% CI 0.569–0.784; log-rank *p* < 0.001) and myocardial infarction (73/6312 [1.2%] vs. 131/6359 [2.1%]; HR 0.555, 95% CI 0.417–0.739; log-rank *p* < 0.001), with no statistically significant differences in cerebral infarction (63/6530 [1.0%] vs. 77/6568 [1.2%]; HR 0.817, 95% CI 0.585–1.139; log-rank *p* = 0.232) or transient ischemic attack (45/6962 [0.6%] vs. 46/6955 [0.7%]; HR 0.969, 95% CI 0.643–1.462; log-rank *p* = 0.883); procedural outcomes again favored incretin-based therapy with fewer cardioversions (72/6390 [1.1%] vs. 175/6491 [2.7%]; HR 0.409, 95% CI 0.311–0.538; log-rank *p* < 0.001) and catheter ablations (66/6972 [0.9%] vs. 107/7154 [1.5%]; HR 0.624, 95% CI 0.459–0.848; log-rank *p* = 0.002), and acute kidney injury remained lower (210/4732 [4.4%] vs. 299/4800 [6.2%]; HR 0.703, 95% CI 0.589–0.838; log-rank *p* < 0.001).

Similarly at 2 years, incretin-based therapy was associated with lower all-cause mortality (658/7593 [8.7%] vs. 829/7592 [10.9%]; HR 0.785, 95% CI 0.708–0.869; log-rank *p* < 0.001), fewer inpatient visits (2646/7079 [37.4%] vs. 3126/7079 [44.2%]; HR 0.782, 95% CI 0.743–0.824; log-rank *p* < 0.001), and fewer ED visits (2449/7079 [34.6%] vs. 2552/7079 [36.1%]; HR 0.944, 95% CI 0.893–0.998; log-rank *p* = 0.042), along with a lower risk of MACE (657/5442 [12.1%] vs. 828/5518 [15.0%]; HR 0.799, 95% CI 0.721–0.885; log-rank *p* < 0.001) and myocardial infarction (184/6312 [2.9%] vs. 299/6359 [4.7%]; HR 0.612, 95% CI 0.509–0.735; log-rank *p* < 0.001), with no statistically significant differences in cerebral infarction (186/6530 [2.8%] vs. 173/6568 [2.6%]; HR 1.079, 95% CI 0.877–1.327; log-rank *p* = 0.473) or transient ischemic attack (123/6962 [1.8%] vs. 115/6955 [1.7%]; HR 1.059, 95% CI 0.821–1.366; log-rank *p* = 0.659); procedural outcomes favored incretin-based therapy with fewer cardioversions (145/6390 [2.3%] vs. 254/6491 [3.9%]; HR 0.560, 95% CI 0.457–0.687; log-rank *p* < 0.001) and catheter ablations (144/6972 [2.1%] vs. 202/7154 [2.8%]; HR 0.715, 95% CI 0.578–0.886; log-rank *p* = 0.002), and acute kidney injury remained lower (501/4732 [10.6%] vs. 652/4800 [13.6%]; HR 0.774, 95% CI 0.689–0.869; log-rank *p* < 0.001). Comparison of outcomes after propensity score matching is shown in Table 2.

### 3.5. Agent-Specific Subgroup Analyses

Agent-specific subgroup analyses were performed to assess whether findings were consistent across the two principal incretin-based agents. In the semaglutide subgroup (3380 matched pairs after 1:1 matching against SGLT2 inhibitors), findings were directionally consistent with the primary analysis across all three follow-up windows: semaglutide was associated with significantly lower all-cause mortality, fewer inpatient visits, and reduced MACE and acute kidney injury compared with SGLT2 inhibitors. Emergency department visit rates did not differ significantly between groups at 1 year (*p* = 0.27). In the tirzepatide subgroup (1245 matched pairs), findings were similarly consistent and directionally favorable for tirzepatide versus SGLT2 inhibitors across primary and secondary outcomes, including lower all-cause mortality, fewer inpatient visits, and reduced MACE; emergency department visits were not significantly different at 1 year (*p* = 0.31). Neither subgroup demonstrated statistically significant differences in ischemic stroke or transient ischemic attack. Detailed outcome data for both subgroups across all follow-up windows are presented in Supplementary Table S6 (semaglutide) and Table S7 (tirzepatide).

## 4. Discussion

HFpEF and AF commonly coexist and potentiate adverse outcomes through shared mechanisms, including elevated filling pressures precipitating left atrial stretch and natriuretic peptide release, systemic and adipose-driven inflammation, endothelial–microvascular dysfunction, and autonomic–neurohormonal activation [11,12,13]. In this context, SGLT2i and incretin-based therapies (GLP-1RA and dual GLP-1/GIP agonists) represent biologically distinct strategies that may differentially influence the HFpEF–AF syndrome. Randomized trial evidence in HFpEF has established SGLT2i as a foundational therapy for reducing worsening HF events, while contemporary HFpEF trials of semaglutide and tirzepatide highlight substantial improvements in health status and functional capacity in obesity-related HFpEF [9,10,14,15].

### 4.1. Mechanistic Considerations: Neurohormonal and Cardiometabolic Modulation in HFpEF and AF

SGLT2i exerts early and sustained cardiorenal effects that are frequently framed as indirect neurohormonal modulation: natriuresis-osmotic diuresis with effective decongestion, improved renal hemodynamics, and downstream reductions in sympathetic and renin–angiotensin–aldosterone system activity [16,17]. These mechanisms align closely with HFpEF pathophysiology characterized by congestion and plausibly mitigate AF-promoting triggers such as atrial stretch and elevated left-sided filling pressures. In HFpEF, recurrent congestion and cardiorenal dysfunction are prime drivers of HF hospitalizations, mitigated by SGTLT2i in multiple trials [18].

In patients with AF, who frequently have higher filling pressures and greater atrial remodeling, hemodynamic unloading may also mitigate AF triggers related to atrial stretch, even if rhythm conversion is not directly targeted [19].

In contrast, GLP-1 receptor agonists and dual GIP/GLP-1 receptor agonists—also known as incretin-based therapies—primarily target cardiometabolic pathways with remodeling and attenuation of systemic inflammatory signaling. The effects are further enhanced by improved insulin resistance and reduced blood pressure. Pathways are particularly salient in obesity-related HFpEF, in which epicardial/visceral adiposity contributes to myocardial and atrial remodeling [20,21]. While GLP-1–based therapies may increase heart rate and are not typically considered neurohormonal antagonists, their upstream adipose and inflammatory effects may indirectly reduce AF substrate and HFpEF symptom burden by lowering hemodynamic parameters and systemic inflammation. The STEP-HFpEF program, enriched for obesity-related HFpEF, provides clinical support for the aforementioned mechanisms, demonstrating robust improvements in symptoms and physical function with semaglutide [22].

### 4.2. Integration and Contextualization of TriNetX Real-World Findings Against Randomized HFpEF Evidence

In EMPEROR-Preserved, empagliflozin reduced the composite of cardiovascular death or HF hospitalization in HFpEF (HR = 0.79), and in DELIVER, dapagliflozin reduced worsening HF or cardiovascular death (HR = 0.82) [14,15]. Importantly, AF is common in HFpEF trials and appears to identify higher baseline risk, yet available subgroup analyses suggest preserved benefit with SGLT2i across AF strata. In DELIVER, dapagliflozin’s efficacy was consistent regardless of baseline AF and examined outcomes across AF subgroups [23]. Similarly, empagliflozin reduced HF outcomes regardless of baseline AF [19]. These RCT data reinforce the current HFpEF management framework that prioritizes SGLT2i as a core disease-modifying therapy [24].

Although our study focused on AF-HFpEF, recent evidence in HFrEF provides additional support for the cardiovascular and antiarrhythmic effects of SGLT2 inhibition. In addition to the established reductions in cardiovascular death and HF hospitalization observed in EMPEROR-Reduced, Mariani et al. recently reported that SGLT2 inhibitor initiation in HFrEF patients with ICD/CRT-D devices was associated with a marked reduction in device-detected atrial and ventricular arrhythmic events over one year. These data suggest that SGLT2 inhibitors may modulate arrhythmogenic substrate through cardiorenal, hemodynamic, neurohormonal, and metabolic mechanisms [25]. Accordingly, our findings should not be interpreted as diminishing the established benefits of SGLT2 inhibitors, but rather as suggesting that incretin-based therapies may confer a phenotype-specific benefit in cardiometabolic AF-HFpEF populations.

Against this backdrop, our TriNetX propensity score–matched analysis (7624 matched pairs; mean age ~71 years; ~52% women; ~60% type 2 diabetes; ~52% overweight/obesity; ~32% CKD) of patients with AF and a HFpEF proxy (I50.3) demonstrated that initiation of incretin-based therapy (GLP-1 receptor agonists and dual GIP/GLP-1 receptor agonists) was associated with lower 1-year all-cause mortality (5.3% vs. 7.3%; HR 0.721, 95% CI 0.634–0.820), fewer inpatient visits (30.0% vs. 37.4%; HR 0.743, 95% CI 0.702–0.787), and non-significantly different emergency department visit rates (27.0% vs. 28.0%; HR 0.946, 95% CI 0.888–1.007; *p* = 0.081) when compared with SGLT2i, with consistent longitudinal findings at 6 months and 2 years.

Importantly, we additionally observed lower risks of MACE (HR = 0.709), myocardial infarction (HR = 0.583), and lower acute kidney injury (AKI) coding in the incretin-based therapy cohort, without statistically significant differences in ischemic stroke or TIA. These findings suggest that within a high-risk AF–HFpEF population managed in real-world practice, incretin-based therapy may be associated with broader reductions in global clinical utilization, morbidity, and mortality than SGLT2i when evaluated using all-cause endpoints. This data aligns with established data regarding GLP-1 agonists in high-risk populations, both with and without diabetes, recorded in the literature, with reductions in MACE and mortality evidenced in the LEADER, SUSTAIN-6, and SELECT trials [26,27,28,29]. Furthermore, our cohort had a reported 60% prevalence of diabetes and comorbid obesity of 52%, explaining the plausibility of improved outcomes seen with GLP-1A over SLGT2i.

Several added considerations may explain these observational findings with the RCT-established HFpEF benefit of SGLT2i. First, the primary utilization outcomes (inpatient visits and ED visits) represent all-cause rather than HF-specific utilization [14]. Additionally, in an older AF–HFpEF cohort (mean age ~71 years) with multiple comorbidities, therapies that substantially reduce adiposity-driven inflammation and improve functional status may exert a broader impact on non-HF utilization and overall mortality than therapies primarily targeting HF congestion biology, particularly if baseline HF event rates are diluted within all-cause utilization. Combining the complex neurohormonal-inflammatory-adiposity-based pathology of HFpEF as described previously, the GLP-1 cardiometabolic targets may aid in decreasing the progression of myocardial stiffness and restrictive physiology and increase total body natriuresis in addition to the benefits observed within SLGT2i. Compounding another complex metabolic and inflammatory comorbidity, such as atrial fibrillation, may further explain the observed association between GLP-1 receptor agonist therapy and lower morbidity in this higher-risk population.

Subsequently, our data aligns with emerging HFpEF incretin trial evidence that is phenotype-specific. Semaglutide in STEP-HFpEF produced large improvements in KCCQ and exercise capacity [10]. Moreover, in a JACC analysis of the STEP-HFpEF program, AF was present in 46% of the participants, supporting the relevance of these findings to HFpEF populations with concomitant AF. Semaglutide improved symptoms, physical limitations, and exercise function, along with reduced weight and inflammatory biomarkers in participants with and without AF and across AF types [30]. SUMMIT demonstrated that tirzepatide reduced the composite of cardiovascular death or worsening HF and improved health status in HFpEF with obesity, extending incretin effects from symptom improvement to reductions in worsening HF events [9].

Our cohort-level associations with reduced mortality and MACE are consistent with the reduction in cardiac and metabolic risk associated with GLP1-RA, with the caveat that direct comparisons of observation and trial cohorts are imperfect. It is important to note that despite adequate propensity score matching across several baseline characteristics, residual variable imbalance, notably BMI, persisted. These factors are strongly associated with both treatment selection and clinical outcomes in HFpEF and may reflect differences in underlying disease severity and metabolic risk. Therefore, the observed associations between incretin-based therapy may partly be explained by residual confounding despite matching.

### 4.3. AF-Related Procedural Outcomes: Signal, Interpretation, and Alternative Explanations

In our cohort, fewer electrical cardioversions (HR = 0.472) and catheter ablations (HR = 0.685) were associated with GLP-1RA compared with SGLT2i at 1 year, with differences present at 6 months and 2 years. This signal could reflect a true reduction in AF symptom burden or progression, consistent with the hypothesis that weight loss and inflammatory cytokine downregulation reduce AF substrate in obesity-related cardiomyopathy, supported by the high AF prevalence and favorable clinical outcomes in the STEP-HFpEF program [22,31]. However, procedural outcomes in EHR datasets are also sensitive to referral patterns, access, operator availability, and multitudes of unmeasured variables.

### 4.4. GLP-1 Receptor Agonism and Combined GIP-GLP-1 Receptor Agonism Interpretation

In our propensity-matched cohort, semaglutide was the most commonly used GLP-1 agonist, administered to 57% of the cohort. Semaglutide when analyzed specifically compared to SGLT2i was significantly associated with lower all-cause mortality at 2 years (HR = 0.701) and composite MACE (HR = 0.753) amongst other outcomes mentioned. These findings are directionally consistent as previously noted in established randomized HFpEF data and represents.

Tirzepatide was the only dual GLP-1/GIP-RA represented in our cohort. When analyzed independently, strong associations with decreases in all-cause mortality (HR = 0.55), MACE (HR 0.75), and Cardioversion (HR = 0.62) were maintained, maintaining the associations observed in the combined cohort. Although direct comparisons between Semaglutide and Tirezpatide head-to-head are out of the scope of this study, when compared with SGLT2i, there are interesting findings when stratifying therapies. Tirzepatide did have a larger overall association in reduction in all-cause mortality at 2-years (HR = 0.55 vs. 0.70) compared to semaglutide. This magnitude difference is consistent with greater weight loss and metabolic effects observed in SURMOUNT-1 and SURMOUNT-5 [32,33].

Semaglutide showed more statistically consistent reductions in MACE, MI, cardioversion, and AKI across time points, likely reflecting the larger cohort size and greater event counts. Tirzepatide, however, showed a numerically stronger association with lower mortality and larger absolute reductions in inpatient visits and AKI at 2 years. This difference may relate to the greater weight loss and metabolic effects typically seen with dual GIP-GLP-1 receptor agonism, which may produce more substantial reductions in visceral adiposity, systemic inflammation, blood pressure, volume overload, and adverse cardiac remodeling. However, these findings should be interpreted as hypothesis-generating rather than definitive evidence of superiority, because the semaglutide and tirzepatide analyses were performed in separate matched cohorts with different sample sizes, baseline risks, and event rates.

### 4.5. Strengths and Limitations

Our study exhibits numerous strengths, incorporating a large multicenter sample from federated EHR networks and monitoring over a long period of time (2 years) with consistent findings across the temporal spectrum. Nonetheless, several important limitations must be acknowledged. First, residual confounding remains a central concern. Despite comorbidity matching, unmeasured differences in HFpEF severity (natriuretic peptides, echocardiographic parameters, functional class), AF burden (paroxysmal vs. persistent, rhythm-control intent), obesity phenotype (BMI, visceral vs. epicardial adiposity), socioeconomic determinants, and medication adherence could influence both treatment selection and outcomes. Notably, even after propensity score matching, meaningful residual imbalances persist in BMI (38.6 vs. 35.6 kg/m^2^; SMD 0.347), BNP (379.6 vs. 622.4 pg/mL; SMD 0.183), HbA1c (7.3 vs. 7.1%; SMD 0.138), indicating persistent confounding by indication that limits causal inference. These differences suggest that patients initiated on incretin-based therapies may be metabolically distinct from those on SGLT2 inhibitors, and all between-group comparisons should be interpreted as associative rather than causal. The TriNetX platform does not support post-matching regression adjustment, precluding additional covariate adjustment for these residual imbalances. Accordingly, residual confounding from these variables cannot be excluded and may have influenced the observed associations. Future studies utilizing patient-level datasets that permit post-matching multivariable adjustment or alternative balancing strategies should seek to address these residual imbalances and further validate these findings. Second, HFpEF phenotyping relied exclusively on ICD-10-CM code I50.3 (diastolic heart failure), without echocardiographic confirmation of preserved ejection fraction or objective structural and functional criteria. Consequently, some patients may have been misclassified, including individuals with HFmrEF or recovered ejection fraction. Such misclassification could have attenuated or biased the observed associations and should be considered when interpreting these findings. Third, the TriNetX platform does not permit identification of disease-specific hospital admissions. Consequently, inpatient visits and emergency department visits as captured in this analysis represent all-cause utilization rather than heart failure-specific or atrial fibrillation-specific hospitalization. Outcomes such as HF hospitalization, AF-related hospitalization, and cardiovascular hospitalization, which would be more clinically aligned with contemporary HFpEF trial endpoints, could not be ascertained. Fourth, MACE in this study incorporates all-cause rather than cardiovascular-specific mortality, as cause-of-death data are not available within TriNetX. This may overestimate the composite endpoint relative to conventional MACE definitions used in randomized trials. Fifth, detailed pharmacological information including drug dose, treatment duration, adherence, and therapy discontinuation is not available in the TriNetX platform. This is particularly relevant given the heterogeneity between agents within the incretin-based therapy cohort (e.g., semaglutide 2.4 mg for weight management vs. 1.0 mg for cardiovascular benefit vs. tirzepatide at varying doses).

## 5. Conclusions

In this propensity-matched EHR analysis, initiation of incretin-based therapy was associated with lower all-cause mortality and healthcare utilization compared with SGLT2i, alongside reductions in MACE, myocardial infarction, and fewer AF-related procedures. These observations complement randomized HFpEF evidence supporting SGLT2i as a foundational therapy for reducing worsening HF events across rhythm strata.

However, residual confounding after propensity matching, limited granularity in HFpEF phenotyping, and treatment-selection bias limit causal interpretation. Prospective randomized studies are needed in specific HFpEF phenotypes, considering AF burden and adjudicated HF-specific endpoints.

## Figures and Tables

**Table 1:** Selected baseline characteristics of the study cohorts before and after propensity score matching.

	Before PSM				After PSM			
	GLP-1/GIP (*n* = 8501)	SGLT2i (*n* = 26,889)	*p*-Value	SMD	GLP-1/GIP (*n* = 7624)	SGLT2i (*n* = 7624)	*p*-Value	SMD
Demographics								
Age at Index	69.8 ± 9.9	76.6 ± 9.6	<0.001	0.702	70.8 ± 9.5	70.8 ± 10.2	0.905	0.002
Female	4489 (52.8%)	13,560 (50.4%)	<0.001	0.048	3972 (52.1%)	3932 (51.6%)	0.517	0.011
Male	4010 (47.2%)	13,291 (49.4%)	<0.001	0.045	3650 (47.9%)	3691 (48.4%)	0.506	0.011
American Indian or Alaska Native	26 (79.1%)	80 (0.3%)	0.903	0.002	22 (0.3%)	23 (0.3%)	0.881	0.002
Diagnosis								
Essential (primary) hypertension	6346 (74.7%)	19,180 (71.3%)	<0.001	0.075	5608 (73.6%)	5566 (73.0%)	0.442	0.012
Type 2 diabetes mellitus	5256 (61.8%)	12,414 (46.2%)	<0.001	0.318	4599 (60.3%)	4748 (62.3%)	0.013	0.04
Overweight and obesity	4668 (54.9%)	9213 (34.3%)	<0.001	0.425	3935 (51.6%)	3952 (51.8%)	0.783	0.004
Chronic kidney disease (CKD)	2590 (30.5%)	10,526 (39.1%)	<0.001	0.183	2435 (31.9%)	2490 (32.7%)	0.341	0.015
Chronic ischemic heart disease	3362 (39.5%)	12,645 (47.0%)	<0.001	0.151	3122 (40.9%)	3137 (41.1%)	0.805	0.004
Acute myocardial infarction	450 (5.3%)	2736 (10.2%)	<0.001	0.184	436 (5.7%)	462 (6.1%)	0.371	0.014
Cerebral infarction	520 (6.1%)	1819 (6.8%)	0.036	0.026	467 (6.1%)	473 (6.2%)	0.84	0.003
Peripheral vascular disease, unspecified	721 (8.5%)	2849 (10.6%)	<0.001	0.072	669 (8.8%)	671 (8.8%)	0.954	0.001
Other chronic obstructive pulmonary disease	1796 (21.1%)	6850 (25.5%)	<0.001	0.103	1666 (21.9%)	1673 (21.9%)	0.891	0.002
Other anemias	1427 (16.8%)	6475 (24.1%)	<0.001	0.182	1331 (17.5%)	1333 (17.5%)	0.966	0.001
Nicotine dependence	636 (7.5%)	2170 (8.1%)	0.08	0.022	580 (7.6%)	582 (7.6%)	0.951	0.001
Medication								
Beta blockers/related	5050 (59.4%)	17,979 (66.9%)	<0.001	0.155	4581 (60.1%)	4601 (60.3%)	0.741	0.005
Diuretics	5216 (61.4%)	20,133 (61.4%)	<0.001	0.293	4791 (62.8%)	4782 (62.7%)	0.88	0.002
Antiarrhythmics	3890 (61.4%)	13,427 (49.9%)	<0.001	0.084	3504 (46.0%)	3493 (45.8%)	0.858	0.003
Ace inhibitors	1647 (19.4%)	4857 (18.1%)	0.007	0.034	1471 (19.3%)	1481 (19.4%)	0.838	0.003
Warfarin	897 (18.1%)	2707 (10.1%)	0.198	0.016	799 (10.5%)	783 (10.3%)	0.671	0.007
Antilipemic agents	5004 (58.9%)	16,029 (58.9%)	0.221	0.015	4486 (58.8%)	4503 (59.1%)	0.780	0.005
Oral hypoglycemic agents, oral	2928 (34.4%)	4868 (18.1%)	<0.001	0.378	2432 (31.9%)	2457 (32.2%)	0.664	0.007
Laboratory								
Natriuretic peptide B [Mass/volume] in Serum, Plasma or Blood	372.3 ± 966.0 (80.2%)	1009.4 ± 2511.9 (80.2%)	<0.001	0.335	379.6 ± 983.0 (20.0%)	622.4 ± 1601.7 (20.4%)	<0.001	0.183
Hemoglobin A1c/total hemoglobin in Blood	7.3 ± 1.8 (62.4%)	7.3 ± 1.8 (48.3%)	<0.001	0.346	7.3 ± 1.8 (60.3%)	7.1 ± 1.6 (60.6%)	<0.001	0.138
BMI	39.1 ± 8.7 (70.5%)	32.0 ± 8.1 (72.4%)	<0.001	0.846	38.6 ± 8.6 (70.2%)	35.6 ± 8.7 (69.8%)	<0.001	0.347
Blood Pressure, Systolic	130.3 ± 18.7 (79.6%)	127.6 ± 20.6 (82.6%)	<0.001	0.14	130.3 ± 18.9 (79.4%)	128.7 ± 20.0 (78.5%)	<0.001	0.083
Blood Pressure, Diastolic	73.1 ± 12.1 (79.6%)	70.0 ± 12.9 (82.6%)	<0.001	0.249	72.8 ± 12.1 (79.4%)	71.8 ± 12.4 (78.5%)	<0.001	0.084
Creatinine [Mass/volume] in Serum, Plasma or Blood	1.2 ± 3.4 (79.6%)	2.4 ± 11.7 (84.6%)	<0.001	0.132	1.3 ± 3.6 (79.3%)	1.4 ± 5.2 (78.9%)	0.115	0.029

Abbreviations: ACE, angiotensin-converting enzyme; BMI, body mass index; CKD, chronic kidney disease; GLP-1, glucagon-like peptide-1; GIP, glucose-dependent insulinotropic polypeptide; PSM, propensity score matching. SGLT2i, sodium-glucose cotransporter-2 inhibitor; SMD, standardized mean difference.

**Table 2:** Comparison of outcomes after propensity score matching.

	Timepoint	Atrial Fibrillation + HFpEF		RD (95% CI)	HR (95% CI)	*p* Value
		GLP1 or GLP1/GIP	SGLT2i			
Primary Outcomes						
All-Cause Mortality	6 months	246/7593 (3.2%)	359/7592 (4.7%)	−0.015 (−0.021, −0.009)	0.677 (0.575, 0.796)	<0.001
	1 year	403/7593 (5.3%)	553/7592 (7.3%)	−0.020 (−0.027, −0.012)	0.721 (0.634, 0.820)	<0.001
	2 years	658/7593 (8.7%)	829/7592 (10.9%)	−0.023 (−0.032, −0.013)	0.785 (0.708, 0.869)	<0.001
Inpatient Visits	6 months	1576/7079 (22.3%)	2101/7079 (29.7%)	−0.074 (−0.089, −0.060)	0.697 (0.653, 0.745)	<0.001
	1 year	2127/7079 (30%)	2684/7079 (37.4%)	−0.074 (−0.089, −0.058)	0.743 (0.702, 0.787)	<0.001
	2 years	2646/7079 (37.4%)	3126/7079 (44.2%)	−0.068 (−0.084, −0.052)	0.782 (0.743, 0.824)	<0.001
Emergency Visits	6 months	1312/7079 (18.5%)	1411/7079 (19.9%)	−0.014 (−0.027, −0.001)	0.911 (0.845, 0.982)	0.015
	1 year	1909/7079 (27%)	1980/7079 (28%)	−0.010 (−0.025, 0.005)	0.946 (0.888, 1.007)	0.081
	2 years	2449/7079 (34.6%)	2552/7079 (36.1%)	−0.015 (−0.030, 0.001)	0.944 (0.893, 0.998)	0.042
Secondary Outcomes						
Cerebral Infarction	6 months	63/6530 (01.0%)	77/6568 (01.2%)	−0.002 (−0.006, 0.001)	0.817 (0.585, 1.139)	0.232
	1 year	106/6530 (01.6%)	123/6568 (01.9%)	−0.002 (−0.007, 0.002)	0.864 (0.666, 1.120)	0.268
	2 years	186/6530 (02.8%)	173/6568 (02.6%)	0.002 (−0.003, 0.008)	1.079 (0.877, 1.327)	0.473
TIA	6 months	45/6962 (00.6%)	46/6955 (00.7%)	−0.000 (−0.003, 0.003)	0.969 (0.643, 1.462)	0.883
	1 year	76/6962 (01.1%)	80/6955 (01.2%)	−0.001 (−0.004, 0.003)	0.943 (0.689, 1.291)	0.715
	2 years	123/6962 (01.8%)	115/6955 (01.7%)	0.001 (−0.003, 0.005)	1.059 (0.821, 1.366)	0.659
MI	6 months	73/6312 (01.2%)	131/6359 (02.1%)	−0.009 (−0.013, −0.005)	0.555 (0.417, 0.739)	<0.001
	1 year	118/6312 (01.9%)	202/6359 (03.2%)	−0.013 (−0.019, −0.008)	0.583 (0.465, 0.732)	<0.001
	2 years	184/6312 (2.9%)	299/6359 (04.7%)	−0.018 (−0.025, −0.011)	0.612 (0.509, 0.735)	<0.001
Composite *MACE	6 months	251/5442 (04.6%)	376/5518 (06.8%)	−0.022 (−0.031, −0.013)	0.668 (0.569, 0.784)	<0.001
	1 year	404/5442 (07.4%)	572/5518 (10.4%)	−0.029 (−0.040, −0.019)	0.709 (0.624, 0.805)	<0.001
	2 years	657/5442 (12.1%)	828/5518 (15.0%)	−0.029 (−0.042, −0.017)	0.799 (0.721, 0.885)	<0.001
Cardioversion	6 months	72/6390 (01.1%)	175/6491 (02.7%)	−0.016 (−0.020, −0.011)	0.409 (0.311, 0.538)	<0.001
	1 year	101/6390 (01.6%)	212/6491 (03.3%)	−0.017 (−0.022, −0.012)	0.472 (0.372, 0.598)	<0.001
	2 years	145/6390 (02.3%)	254/6491 (03.9%)	−0.016 (−0.022, −0.010)	0.560 (0.457, 0.687)	<0.001
AF Ablation	6 months	66/6972 (00.9%)	107/7154 (01.5%)	−0.005 (−0.009, −0.002)	0.624 (0.459, 0.848)	0.002
	1 year	105/6972 (01.5%)	155/7154 (02.2%)	−0.007 (−0.011, −0.002)	0.685 (0.534, 0.877)	0.003
	2 years	144/6972 (02.1%)	202/7154 (02.8%)	−0.008 (−0.013, −0.002)	0.715 (0.578, 0.886)	0.002
AKI	6 months	210/4732 (04.4%)	299/4800 (06.2%)	−0.018 (−0.027, −0.009)	0.703 (0.589, 0.838)	<0.001
	1 year	330/4732 (07.0%)	440/4800 (09.2%)	−0.022 (−0.033, −0.011)	0.751 (0.652, 0.867)	<0.001
	2 years	501/4732 (10.6%)	652/4800 (13.6%)	−0.030 (−0.043, −0.017)	0.774 (0.689, 0.869)	<0.001

Abbreviations: RD, risk difference; HR, Hazard Ratio; CI, confidence interval; MACE, major adverse cardiovascular events; AF, atrial fibrillation; AKI, acute kidney injury; MI, myocardial infarction; TIA, transient ischemic attack; HFpEF, Heart Failure with preserved Ejection Fraction. *MACE is a composite outcome including mortality, ischemic stroke, and acute myocardial infarction.

## Data Availability

The data that support the findings of this study are available from the TriNetX Research Network. Due to licensing restrictions and data use agreements, the raw data are not publicly available. Access to the TriNetX platform can be obtained through institutional subscription. Aggregate data supporting the findings of this study may be available from the corresponding author upon reasonable request and with permission from TriNetX.

## Supplementary Materials

The following supporting information can be downloaded at: https://www.mdpi.com/article/10.3390/jcm15134992/s1, Supplementary Table S1: Participants eligibility with the relevant used codes; Supplementary Table S2: Baseline selected variables with their relevant codes; Table S3: Outcome definition and codes used for identification; Table S4: Detailed baseline characteristics of the study cohort before and after propensity score matching; Table S5: Distribution of individual agents in the GLP-1 receptor agonist/dual GIP/GLP-1 receptor agonist cohort and SGLT2 inhibitor cohort; Table S6: Comparison of outcomes after propensity score matching for Semaglutide Subgroup; Table S7: Comparison of outcomes after propensity score matching for Tirzepatide Subgroup.
