# Supplementary material for: GLP-1 Receptor Agonists or Dual GLP-1/GIP Receptor Agonists vs. SGLT2 Inhibitors in Patients with Atrial Fibrillation and HFpEF: A Propensity-Matched Real-World Analysis
Source: J Clin Med. 2026 Jun 26;15(13):4992. doi: 10.3390/jcm15134992 (PMC13362885; doi:10.3390/jcm15134992)
Supplement: Supplementary file 1 [file jcm-15-04992-s001.zip › jcm-4333902-supplementary.pdf]

Supplementary Material

Table S1: Participants eligibility with the relevant used codes

Table S2: Baseline selected variables with their relevant codes

Table S3: Outcome definition and codes used for identification

Table S4: Detailed baseline characteristics of the study cohort before and after propensity score matching

Table S5: Distribution of individual agents in the GLP-1 receptor agonist/dual GIP/GLP-1 receptor agonist cohort and SGLT2 inhibitor cohort

Table S6: Comparison of outcomes after propensity score matching for Semaglutide Subgroup (Table S6)

Table S7: Comparison of outcomes after propensity score matching for Tirzepatide Subgroup (Table S7)

Table S1: Participants eligibility with the relevant used codes

Cohort 1: GLP-1 Receptor Agonists and Dual GIP/GLP-1 Receptor Agonists

Cohort 2: SGLT2i

Cohort 1: GLP-1 Receptor Agonists and Dual GIP/GLP-1 Receptor Agonists

**Group 1**				
**Group 1A AF and HFpEF**				
must have		diagnosis	UMLS:ICD10CM:I50.3	Diastolic (congestive) heart failure
	and	diagnosis	UMLS:ICD10CM:I48	Atrial fibrillation and flutter
cannot have		diagnosis	UMLS:ICD10CM:I50.2	Systolic (congestive) heart failure
	or	diagnosis	UMLS:ICD10CM:Z94.1	Heart transplant status
	or	diagnosis	UMLS:ICD10CM:Z95.811	Presence of heart assist device
	or	diagnosis	UMLS:ICD10CM:O00-O9A	Pregnancy, childbirth and the puerperium
	or	diagnosis	UMLS:ICD10CM:N18.6	End stage renal disease
	or	diagnosis	UMLS:ICD10CM:Z99.2	Dependence on renal dialysis
	or	diagnosis	UMLS:ICD10CM:Z49	Encounter for care involving renal dialysis
date constraint		The terms in this group occurred on or before Jan 1, 2024		
event relationship		The first instance of GLP-1 Receptor Agonists and Dual GIP/GLP-1 Receptor Agonists occurred within 1 month on or after any instance of AF and HFpEF		
**Group 1B GLP-1 Receptor Agonists and Dual GIP/GLP-1 Receptor Agonists**				
must have	any of	medication	NLM:RXNORM:1440051	lixisenatide
		medication	NLM:RXNORM:2601723	tirzepatide
		medication	NLM:RXNORM:1991302	semaglutide
		medication	NLM:RXNORM:475968	liraglutide
		medication	NLM:RXNORM:60548	exenatide
		medication	NLM:RXNORM:1534763	albiglutide
		medication	NLM:RXNORM:1551291	dulaglutide
cannot have		medication	NLM:RXNORM:1545653	empagliflozin
	or	medication	NLM:RXNORM:1488564	dapagliflozin
	or	medication	NLM:RXNORM:1373458	canagliflozin
	or	medication	NLM:RXNORM:1992672	ertugliflozin
	or	medication	NLM:RXNORM:2638675	sotagliflozin

Cohort 2: SGLT2i

**Group 1**				
**Group 1A AF and HFpEF**				
must have		diagnosis	UMLS:ICD10CM:I50.3	Diastolic (congestive) heart failure
	and	diagnosis	UMLS:ICD10CM:I48	Atrial fibrillation and flutter
cannot have		diagnosis	UMLS:ICD10CM:I50.2	Systolic (congestive) heart failure
	or	diagnosis	UMLS:ICD10CM:Z94.1	Heart transplant status
	or	diagnosis	UMLS:ICD10CM:Z95.811	Presence of heart assist device
	or	diagnosis	UMLS:ICD10CM:O00-O9A	Pregnancy, childbirth and the puerperium
	or	diagnosis	UMLS:ICD10CM:N18.6	End stage renal disease
	or	diagnosis	UMLS:ICD10CM:Z99.2	Dependence on renal dialysis
	or	diagnosis	UMLS:ICD10CM:Z49	Encounter for care involving renal dialysis
date constraint		The terms in this group occurred on or before Jan 1, 2024		
event relationship		The first instance of SGLT2i occurred within 1 month on or after any instance of AF and HFpEF		
**Group 1B SGLT2i**				
must have	any of	medication	NLM:RXNORM:1545653	empagliflozin
		medication	NLM:RXNORM:1992672	ertugliflozin
		medication	NLM:RXNORM:2638675	sotagliflozin
		medication	NLM:RXNORM:1373458	canagliflozin
		medication	NLM:RXNORM:1488564	dapagliflozin
cannot have		medication	NLM:RXNORM:1440051	lixisenatide
	or	medication	NLM:RXNORM:2601723	tirzepatide
	or	medication	NLM:RXNORM:475968	liraglutide
	or	medication	NLM:RXNORM:1991302	semaglutide
	or	medication	NLM:RXNORM:60548	exenatide
	or	medication	NLM:RXNORM:1534763	albiglutide
	or	medication	NLM:RXNORM:1551291	dulaglutide

Table S2: Baseline selected variables with their relevant codes

Category	Code	Description
**Demographics**	AI	Age at Index
	F	Female
	2054-5	Black or African American
	M	Male
	2106-3	White
	1002-5	American Indian or Alaska Native
	UNK	Unknown Race
	2076-8	Native Hawaiian or Other Pacific Islander
	UN	Unknown Ethnicity
	2186-5	Not Hispanic or Latino
	2135-2	Hispanic or Latino
	2131-1	Other Race
	2028-9	Asian
**Diagnosis**	I10	Essential (primary) hypertension
	E11	Type 2 diabetes mellitus
	E66	Overweight and obesity
	N18	Chronic kidney disease (CKD)
	N17	Acute kidney failure
	I25	Chronic ischemic heart disease
	I21	Acute myocardial infarction
	I63	Cerebral infarction
	G45	Transient cerebral ischemic attacks and related syndromes
	I70	Atherosclerosis

	I73.9	Peripheral vascular disease, unspecified
	J44	Other chronic obstructive pulmonary disease
	G47.33	Obstructive sleep apnea (adult) (pediatric)
	D50	Iron deficiency anemia
	D64	Other anemias
	K74	Fibrosis and cirrhosis of liver
	K70	Alcoholic liver disease
	C00-D49	Neoplasms
	Z72.0	Tobacco use
	Z87.891	Personal history of nicotine dependence
	F10	Alcohol related disorders
	F17	Nicotine dependence
**Medication**	CV100	BETA BLOCKERS/RELATED
	CV700	DIURETICS
	CV300	ANTIARRHYTHMICS
	CV800	ACE INHIBITORS
	11289	warfarin
	1114195	rivaroxaban
	1364430	apixaban
	1037042	dabigatran etexilate
	1191	aspirin
	32968	clopidogrel
	CV805	ANGIOTENSIN II INHIBITOR
	69749	valsartan
		sacubitril

	1656328	
	CV350	ANTILIPEMIC AGENTS
	HS502	ORAL HYPOGLYCEMIC AGENTS, ORAL
**Laboratory**	9014	Hemoglobin [Mass/volume] in Blood
	9003	Natriuretic peptide B [Mass/volume] in Serum, Plasma or Blood
	9072	Natriuretic peptide.B prohormone N-Terminal [Mass/volume] in Serum, Plasma or Blood
	9037	Hemoglobin A1c/Hemoglobin.total in Blood
	9083	BMI
	9085	Blood Pressure, Systolic
	9086	Blood Pressure, Diastolic
	9024	Creatinine [Mass/volume] in Serum, Plasma or Blood

Table S3: Outcomes definition and codes used for identification

**All-Cause Mortaity**			
**Outcome definition**			
	Demographics	Deceased	Deceased
**Settings for the performed analyses**			
	Kaplan - Meier survival analysis		excluding patients with outcome prior to the time window
	Risk analysis		excluding patients with outcome prior to the time window
	Number of instances analysis		excluding patients with outcome prior to the time window<br>including patients with zero outcomes<br>counts are grouped by date
**Inpatient Visit**			
**Outcome definition**			
	Visit	UMLS:HL7V3.0:VisitType:ACUTE	Visit: Inpatient Acute
	Visit	UMLS:HL7V3.0:VisitType:IMP	Visit: Inpatient Encounter
	Visit	UMLS:HL7V3.0:VisitType:NONAC	Visit: Inpatient Non-acute
**Settings for the performed analyses**			
	Kaplan - Meier survival analysis		including patients with outcome prior to the time window
	Number of instances analysis		including patients with outcome prior to the time window<br>including patients with zero outcomes<br>counts are grouped by date
	Risk analysis		including patients with outcome prior to the time window
**Cerebral Infarction**			
**Outcome definition**			
	Diagnosis	UMLS:ICD10CM:I63	Cerebral infarction
**Settings for the performed analyses**			
	Kaplan - Meier survival analysis		excluding patients with outcome prior to the time window
	Risk analysis		excluding patients with outcome prior to the time window
	Number of instances analysis		excluding patients with outcome prior to the time window<br>including patients with zero outcomes<br>counts are grouped by date
**TIA**			
**Outcome definition**			
	Diagnosis	UMLS:ICD10CM:G45	Transient cerebral ischemic attacks and related syndromes
**Settings for the performed analyses**			
	Kaplan - Meier survival analysis		excluding patients with outcome prior to the time window
	Risk analysis		excluding patients with outcome prior to the time window
	Number of instances analysis		excluding patients with outcome prior to the time window<br>including patients with zero outcomes<br>counts are grouped by date
**MI**			
**Outcome definition**			
	Diagnosis	UMLS:ICD10CM:I21	Acute myocardial infarction
**Settings for the performed analyses**			
	Risk analysis		excluding patients with outcome prior to the time window
	Number of instances analysis		excluding patients with outcome prior to the time window<br>including patients with zero outcomes<br>counts are grouped by date
	Kaplan - Meier survival analysis		excluding patients with outcome prior to the time window
**Composite MACE**			
**Outcome definition**			
	Demographics	Deceased	Deceased
	Diagnosis	UMLS:ICD10CM:I21	Acute myocardial infarction
	Diagnosis	UMLS:ICD10CM:I63	Cerebral infarction

**Settings for the performed analyses**		
Number of instances analysis		excluding patients with outcome prior to the time window<br>including patients with zero outcomes<br>counts are grouped by date
Risk analysis		excluding patients with outcome prior to the time window
Kaplan - Meier survival analysis		excluding patients with outcome prior to the time window
**Cardioversion**		
**Outcome definition**		
Procedure	UMLS:CPT:92960	Cardioversion, elective, electrical conversion of arrhythmia; external
Procedure	UMLS:CPT:92961	Cardioversion, elective, electrical conversion of arrhythmia; internal (separate procedure)
**Settings for the performed analyses**		
Risk analysis		excluding patients with outcome prior to the time window
Kaplan - Meier survival analysis		excluding patients with outcome prior to the time window
Number of instances analysis		excluding patients with outcome prior to the time window<br>including patients with zero outcomes<br>counts are grouped by date
**AF Ablation**		
**Outcome definition**		
Procedure	UMLS:CPT:93656	Comprehensive electrophysiologic evaluation including transseptal catheterizations, insertion and repositioning of multiple electrode catheters with intracardiac catheter ablation of atrial fibrillation by pulmonary vein isolation, including intracardiac electrophysiologic 3-dimensional mapping, intracardiac echocardiography including imaging supervision and interpretation, induction or attempted induction of an arrhythmia including left or right atrial pacing/recording, right ventricular pacing/recording, and His bundle recording, when performed
**Settings for the performed analyses**		
Risk analysis		excluding patients with outcome prior to the time window
Kaplan - Meier survival analysis		excluding patients with outcome prior to the time window
Number of instances analysis		excluding patients with outcome prior to the time window<br>including patients with zero outcomes<br>counts are grouped by date
**AKI**		
**Outcome definition**		
Diagnosis	UMLS:ICD10CM:N17	Acute kidney failure
**Settings for the performed analyses**		
Number of instances analysis		excluding patients with outcome prior to the time window<br>including patients with zero outcomes<br>counts are grouped by date
Kaplan - Meier survival analysis		excluding patients with outcome prior to the time window
Risk analysis		excluding patients with outcome prior to the time window
**Emergency Visit**		
**Outcome definition**		
Visit	UMLS:HL7V3.0:VisitType:EMER	Visit: Emergency
**Settings for the performed analyses**		
Risk analysis		including patients with outcome prior to the time window
Number of instances analysis		including patients with outcome prior to the time window<br>including patients with zero outcomes<br>counts are grouped by date
Kaplan - Meier survival analysis		including patients with outcome prior to the time window

Table S4: Detailed baseline characteristics of the study cohort before and after propensity score matching

	Before PSM				After PSM			
	GLP1/GIP (n=8,501)	SGLT2i (n=26,889)	p-Value	SMD	GLP1/GIP (n=7,624)	SGLT2i (n=7,624)	p-Value	SMD
Demographics								
Age at Index	69.8 +/- 9.9	76.6 +/- 9.6	<0.001	0.702	70.8 +/- 9.5	70.8 +/- 10.2	0.905	0.002
Female	4489 (52.8%)	13560 (50.4%)	<0.001	0.048	3972 (52.1%)	3932 (51.6%)	0.517	0.011
Black or African American	972 (11.4%)	2862 (10.6%)	0.041	0.025	859 (11.3%)	852 (11.2%)	0.857	0.003
Male	4010 (47.2%)	13291 (49.4%)	<0.001	0.045	3650 (47.9%)	3691 (48.4%)	0.506	0.011
White	6944 (81.7%)	21281 (79.1%)	<0.001	0.064	6231 (81.7%)	6220 (81.6%)	0.818	0.004
American Indian or Alaska Native	26 (79.1%)	80 (0.3%)	0.903	0.002	22 (0.3%)	23 (0.3%)	0.881	0.002
Unknown Race	234 (2.8%)	1186 (4.4%)	<0.001	0.089	213 (2.8%)	219 (2.9%)	0.77	0.005
Native Hawaiian or Other Pacific Islander	50 (0.6%)	146 (0.5%)	0.625	0.006	43 (0.6%)	41 (0.5%)	0.827	0.004
Unknown Ethnicity	1521 (17.9%)	4680 (17.4%)	0.303	0.013	1356 (17.8%)	1367 (17.9%)	0.816	0.004
Not Hispanic or Latino	6696 (78.8%)	21300 (79.2%)	0.377	0.011	6017 (78.9%)	5990 (78.6%)	0.593	0.009
Hispanic or Latino	284 (3.3%)	909 (3.4%)	0.859	0.002	251 (3.3%)	267 (3.5%)	0.474	0.012
Other Race	157 (1.8%)	539 (2.0%)	0.361	0.011	142 (1.9%)	150 (2.0%)	0.636	0.008
Asian	118(1.4%)	795 (3.0%)	<0.001	0.108	114 (1.5%)	119 (1.6%)	0.741	0.005
Diagnosis								
Essential (primary) hypertension	6346 (74.7%)	19180 (71.3%)	<0.001	0.075	5608 (73.6%)	5566 (73.0%)	0.442	0.012
Type 2 diabetes mellitus	5256 (61.8%)	12414 (46.2%)	<0.001	0.318	4599 (60.3%)	4748 (62.3%)	0.013	0.04
Overweight and obesity	4668 (54.9%)	9213 (34.3%)	<0.001	0.425	3935 (51.6%)	3952 (51.8%)	0.783	0.004
Chronic kidney disease (CKD)	2590 (30.5%)	10526 (39.1%)	<0.001	0.183	2435 (31.9%)	2490 (32.7%)	0.341	0.015

Acute kidney failure	1287 (15.1%)	6581 (24.5%)	<0.001	0.236	1239 (16.3%)	1293 (17.0%)	0.24	0.019
Chronic ischemic heart disease	3362 (39.5%)	12645 (47.0%)	<0.001	0.151	3122 (40.9%)	3137 (41.1%)	0.805	0.004
Acute myocardial infarction	450 (5.3%)	2736 (10.2%)	<0.001	0.184	436 (5.7%)	462 (6.1%)	0.371	0.014
Cerebral infarction	520 (6.1%)	1819 (6.8%)	0.036	0.026	467 (6.1%)	473 (6.2%)	0.84	0.003
Transient cerebral ischemic attacks and related syndromes	209 (2.5%)	801 (3.0%)	0.012	0.032	194 (2.5%)	200 (2.6%)	0.759	0.005
Atherosclerosis	710 (8.4%)	2989 (11.1%)	<0.001	0.093	670 (8.8%)	667 (8.7%)	0.932	0.001
Peripheral vascular disease, unspecified	721 (8.5%)	2849 (10.6%)	<0.001	0.072	669 (8.8%)	671 (8.8%)	0.954	0.001
Other chronic obstructive pulmonary disease	1796 (21.1%)	6850 (25.5%)	<0.001	0.103	1666 (21.9%)	1673 (21.9%)	0.891	0.002
Obstructive sleep apnea (adult) (pediatric)	3510 (41.3%)	7697 (28.6%)	<0.001	0.268	2986 (39.2%)	2962 (38.9%)	0.69	0.006
Iron deficiency anemia	1008 (11.9%)	4386 (16.3%)	<0.001	0.128	940 (12.3%)	950 (12.5%)	0.806	0.004
Other anemias	1427 (16.8%)	6475 (24.1%)	<0.001	0.182	1331 (17.5%)	1333 (17.5%)	0.966	0.001
Fibrosis and cirrhosis of liver	260 (3.1%)	1004 (3.7%)	0.003	0.037	243 (3.2%)	240 (3.1%)	0.89	0.002
Alcoholic liver disease	43 (0.5%)	251 (0.9%)	<0.001	0.051	42 (0.6%)	42 (0.6%)	1	0.001
Neoplasms	2044 (24.0%)	6785 (25.2%)	0.027	0.028	1814 (23.8%)	1794 (23.5%)	0.703	<0.001
Tobacco use	167 (2.0%)	639 (2.4%)	0.026	0.028	151 (2.0%)	148 (1.9%)	0.861	0.003
Personal history of nicotine dependence	2056 (24.2%)	7624 (28.4%)	<0.001	0.095	1899 (24.9%)	1895 (24.9%)	0.94	0.001
Alcohol related disorders	252 (3.0%)	956 (3.6%)	0.009	0.033	230 (3.0%)	211 (2.8%)	0.359	0.015
Nicotine dependence	636 (7.5%)	2170 (8.1%)	0.08	0.022	580 (7.6%)	582 (7.6%)	0.951	0.001
Medication								
BETA BLOCKERS/RELATED	5050 (59.4%)	17979 (66.9%)	<0.001	0.155	4581 (60.1%)	4601 (60.3%)	0.741	0.005
DIURETICS	5216 (61.4%)	20133 (61.4%)	<0.001	0.293	4791 (62.8%)	4782 (62.7%)	0.88	0.002

ANTIARRHYTHMICS	3890 (61.4%)	13427 (49.9%)	<0.001	0.084	3504 (46.0%)	3493 (45.8%)	0.858	0.003
ACE INHIBITORS	1647 (19.4%)	4857 (18.1%)	0.007	0.034	1471 (19.3%)	1481 (19.4%)	0.838	0.003
Warfarin	897 (18.1%)	2707 (10.1%)	0.198	0.016	799 (10.5%)	783 (10.3%)	0.671	0.007
Rivaroxaban	921 (10.8%)	2832 (10.5%)	0.431	0.01	821 (10.8%)	822 (10.8%)	0.979	<0.001
Apixaban	2759 (32.5%)	11229 (41.8%)	<0.001	0.194	2528 (33.2%)	2552 (33.5%)	0.680	0.007
Dabigatran Etexilate	101 (1.2%)	323 (1.2%)	0.923	0.001	91 1.2%	90 (1.2%)	0.940	0.001
Aspirin	2183 (25.7%)	8728 (32.5%)	<0.001	0.15	2025 (26.6%)	2106 (27.6%)	0.140	0.024
Clopidogrel	747 (8.8%)	2719 (10.1%)	<0.001	0.045	679 (8.9%)	701 (9.2%)	0.535	0.01
ANGIOTENSIN II INHIBITOR	2189 (25.7%)	7743 (28.8%)	<0.001	0.068	1962 (25.7%)	1977 (25.9%)	0.781	0.004
Valsartan	460 (5.4%)	2319 (8.6%)	<0.001	0.126	421 (5.5%)	428 (5.6%)	0.805	0.004
Sacubitril	106 (1.2%)	1151 (4.3%)	<0.001	0.186	103 (1.4%)	95 (1.2%)	0.567	0.009
ANTILIPEMIC AGENTS	5004 (58.9%)	16029 (58.9%)	0.221	0.015	4486 (58.8%)	4503 (59.1%)	0.780	0.005
ORAL HYPOGLYCEMIC AGENTS,ORAL	2928 (34.4%)	4868 (18.1%)	<0.001	0.378	2432 (31.9%)	2457 (32.2%)	0.664	0.007
Laboratory								
Hemoglobin [Mass/volume] in Blood	12.7 +/- 2.0 (73.2%)	11.9 +/- 2.3 (73.2%)	<0.001	0.377	12.7 +/- 2.0 (73.4%)	12.4 +/- 2.2 (73.5%)	<0.001	0.140
Natriuretic peptide B [Mass/volume] in Serum, Plasma or Blood	372.3 +/- 966.0 (80.2%)	1009.4 +/- 2511.9 (80.2%)	<0.001	0.335	379.6 +/- 983.0 (20.0%)	622.4 +/- 1601.7 (20.4%)	<0.001	0.183
Natriuretic peptide.B prohormone N-Terminal [Mass/volume] in Serum, Plasma or Blood	1463.7 +/- 2521.4 (14.9%)	3414.9 +/- 5117.6 (28.0%)	<0.001	0.484	1492.0 +/- 2558.9 (16.0%)	2426.5 +/- 4092.7 (16.4%)	<0.001	0.274
Hemoglobin A1c/Hemoglobin.total in Blood	7.3 +/- 1.8 (62.4%)	7.3 +/- 1.8 (48.3%)	<0.001	0.346	7.3 +/- 1.8 (60.3%)	7.1 +/- 1.6 (60.6%)	<0.001	0.138

BMI	39.1 +/- 8.7 (70.5%)	32.0 +/- 8.1 (72.4%)	<0.001	0.846	38.6 +/- 8.6 (70.2%)	35.6 +/- 8.7 (69.8%)	<0.001	0.347
Blood Pressure, Systolic	130.3 +/- 18.7 (79.6%)	127.6 +/- 20.6 (82.6%)	<0.001	0.14	130.3 +/- 18.9 (79.4%)	128.7 +/- 20.0 (78.5%)	<0.001	0.083
Blood Pressure, Diastolic	73.1 +/- 12.1 (79.6%)	70.0 +/- 12.9 (82.6%)	<0.001	0.249	72.8 +/- 12.1 (79.4%)	71.8 +/- 12.4 (78.5%)	<0.001	0.084
Creatinine [Mass/volume] in Serum, Plasma or Blood	1.2 +/- 3.4 (79.6%)	2.4 +/- 11.7 (84.6%)	<0.001	0.132	1.3 +/- 3.6 (79.3%)	1.4 +/- 5.2 (78.9%)	0.115	0.029

ACE, angiotensin-converting enzyme; ARB, angiotensin II receptor blocker; BMI, body mass index; BNP, B-type natriuretic peptide.
CKD, chronic kidney disease; GLP-1, glucagon-like peptide-1; GIP, glucose-dependent insulinotropic polypeptide; HbA1c, hemoglobin A1c.
MI, myocardial infarction; NT-proBNP, N-terminal pro–B-type natriuretic peptide; OSA, obstructive sleep apnea; PSM, propensity score matching.
SGLT2i, sodium-glucose cotransporter-2 inhibitor; SMD, standardized mean difference.

Table S5: Distribution of individual agents in the GLP-1 receptor agonist/dual GIP/GLP-1 receptor agonist cohort and SGLT2 inhibitor cohort

This table presents the distribution of individual pharmacological agents within each cohort.

GLP-1 Receptor Agonists	
Semaglutide	57%
Dulaglutide	26%
Liraglutide	11%
Exenatide	3%
Albiglutide	0%
Lixisenatide	1%
Dual GIP/GLP-1 Receptor Agonist	
Tirzepatide	28%
SGLT2 Inhibitors	
Empagliflozin	73%
Dapagliflozin	36%
Canagliflozin	2%
Ertugliflozin	0%
Sotagliflozin	0%

Abbreviations: GIP, glucose-dependent insulinotropic polypeptide; GLP-1, glucagon-like peptide-1; RA, receptor agonist; SGLT2i, sodium-glucose cotransporter-2 inhibitor.

Table S6: Comparison of outcomes after propensity score matching for Semaglutide Subgroup (Table S6)

	Timepoint	Atrial Fibrillation + HFpEF		RD (95% CI)	HR (95% CI)	P value
		Semaglutide	SGLT2i			
Primary Outcomes						
**All-Cause Mortality**	6 months	95/3,380 (2.8%)	166/3371 (4.9%)	-0.021 (-0.030, -0.012)	0.561 (0.436, 0.721)	<0.001
	1 year	165/3380 (4.9%)	260/3371 (7.7%)	-0.028 (-0.040, -0.017)	0.625 (0.514, 0.759)	<0.001
	2 years	276/3,380 (8.2%)	396/3,371 (11.7%)	-0.036 (-0.050, -0.022)	0.701 (0.601, 0.817)	<0.001
**Inpatient Visits**	6 months	729/3,393 (21.5%)	987/3393 (29.1%)	-0.076 (-0.097, -0.055)	0.683 (0.621, 0.751)	<0.001
	1 year	1004/3393 (29.6%)	1293/3393 (36.5%)	-0.069 (-0.092, -0.047)	0.748 (0.688, 0.813)	<0.001
	2 years	1251/3393 (36.9%)	1472/3393 (43.4%)	-0.065 (-0.088, -0.042)	0.795 (0.737, 0.857)	<0.001
**Emergency Visits**	6 months	611/3,393 (18.0%)	653/3,393 (19.2%)	-0.012 (-0.031, 0.006)	0.916 (0.820, 1.023)	0.119
	1 year	905/3,393 (26.7%)	936/3,393 (27.6%)	-0.009 (-0.030, 0.012)	0.950 (0.867, 1.041)	0.27
	2 years	1155/3,393 (34.0%)	1217/3,393 (35.9%)	-0.018 (-0.041, 0.004)	0.946 (0.873, 1.026)	0.180
Secondary Outcomes						
**Cerebral Infarction**	6 months	29/2866 (1.0%)	46/2939 (1.6%)	-0.006 (-0.011, 0.000)	0.638 (0.401, 1.016)	0.056
	1 year	52/2866 (1.8%)	68/2939 (2.3%)	-0.005 (-0.012, 0.002	0.781 (0.544, 1.121)	0.179
	2 years	79/2866 (2.8%)	103/2939 (3.5%)	-0.007 (-0.016, 0.001)	0.797 (0.595, 1.069)	0.129
**TIA**	6 months	28/3031 (0.9%)	20/3,102 (0.6%)	0.003 (-0.002, 0.007)	1.419 (0.800, 2.519)	0.229

	1 year	43/3,031 (1.4%)	35/3,102 (1.1%)	0.003 (-0.003, 0.009)	1.254 (0.802, 1.959)	0.32
	2 years	63/3,031 (2.1%)	56/3,102 (1.8%)	0.003 (-0.004, 0.010)	1.169 (0.815, 1.675)	0.396
**MI**	6 months	28/2,750 (1.0%)	49/2,754 (1.8%)	-0.008 (-0.014, -0.001)	0.564 (0.355, 0.898)	0.014
	1 year	58/2,750 (2.1%)	77/2,754 (2.8%)	-0.007 (-0.015, 0.001)	0.750 (0.534, 1.055)	0.097
	2 years	91/2,750 (3.3%)	126/2,754 (4.6%)	-0.013 (-0.023, -0.002)	0.735 (0.561, 0.962)	0.024
**Composite *MACE**	6 months	97/2,364 (4.1%)	173/2,398 (7.2%)	-0.031 (-0.044, -0.018)	0.558 (0.435, 0.715)	<0.001
	1 year	175/2,364 (7.4%)	254/2,398 (10.6%)	-0.032 (-0.048, -0.016)	0.691 (0.570, 0.837)	<0.001
	2 years	280/2,364 (11.8%)	381/2,398 (15.9%)	-0.040 (-0.060, -0.021)	0.753 (0.645, 0.879)	<0.001
**Cardioversion**	6 months	19/2,839 (0.7%)	67/2,906 (2.3%)	-0.016 (-0.023, -0.010)	0.284 (0.171, 0.473)	<0.001
	1 year	31/2,839 (1.1%)	87/2,906 (3.0%)	-0.019 (-0.026, -0.012)	0.357 (0.237, 0.538)	<0.001
	2 years	57/2,839 (2.0%)	106/2,906 (3.6%)	-0.016 (-0.025, -0.008)	0.546 (0.396, 0.753)	<0.001
**AF Ablation**	6 months	28/3,083 (0.9%)	40/3,182 (1.3%)	-0.003 (-0.009, 0.002	0.711 (0.439, 1.153)	0.165
	1 year	42/3,083 (1.4%)	62/3,182 (1.9%)	-0.006 (-0.012, 0.000)	0.691 (0.467, 1.023)	0.063
	2 years	65/3,083 (2.1%)	79/3,182 (2.5%)	-0.004 (-0.011, 0.004)	0.851 (0.613, 1.182)	0.335
**AKI**	6 months	84/2,130 (3.9%)	147/2,237 (6.6%)	-0.026 (-0.039, -0.013)	0.584 (0.446, 0.763)	<0.001
	1 year	140/2,130 (6.6%)	211/2,237 (9.4%)	-0.029 (-0.045, -0.013)	0.679 (0.548, 0.840)	<0.001
	2 years	217/2,130 (10.2%)	302/2,237 (13.5%)	-0.033 (-0.052, -0.014)	0.744 (0.625, 0.886)	<0.001

Abbreviations: OACs = oral anticoagulants; RD = risk difference; HR= Hazard Ratio; CI = confidence interval; MACE = major adverse cardiovascular events.

** MACE* is a composite outcome including **mortality, ischemic stroke, and acute myocardial infarction**.

AF, atrial fibrillation; AKI, acute kidney injury; ED, emergency department; MACE,MI, myocardial infarction; TIA, transient ischemic attack.

HFpEF = Heart Failure with preserved Ejection Fraction

Table S7: Comparison of outcomes after propensity score matching for Tirzepatide Subgroup

	Timepoint	Atrial Fibrillation + HFpEF		RD (95% CI)	HR (95% CI)	P value
		Tirzepatide	SGLT2i			
Primary Outcomes						
**All-Cause Mortality**	6 months	17/1,245 (1.4%)	38/1,245 (3.1%)	-0.017 (-0.028, -0.005)	0.477 (0.269, 0.845)	0.009
	1 year	28/1,245 (2.2%)	71/1,245 (5.7%)	-0.035 (-0.050, -0.019)	0.458 (0.296, 0.710)	<0.001
	2 years	45/1,245 (3.6%)	113/1,245 (9.1%)	-0.055 (-0.074, -0.036)	0.552 (0.390, 0.782)	<0.001
**Inpatient Visits**	6 months	266/1,251 (21.3%)	356/1,251 (28.5%)	-0.072 (-0.106, -0.038)	0.738 (0.629, 0.865)	<0.001
	1 year	337/1,251 (26.9%)	441/1,251 (35.3%)	-0.083 (-0.119, -0.047)	0.788 (0.684, 0.908)	<0.001
	2 years	391/1,251 (31.3%)	523/1251 (41.8%)	-0.106 (-0.143, -0.068)	0.844 (0.739, 0.963)	0.012
**Emergency Visits**	6 months	193/1,251 (15.4%)	217/1,251 (17.3%)	-0.019 (-0.048, 0.010)	0.938 (0.773, 1.139)	0.518
	1 year	261/1,251 (20.9%)	318/1,251 (25.4%)	-0.046 (-0.079, -0.013)	0.919 (0.780, 1.083)	0.314
	2 years	323/1,251 (25.8%)	404/1,251 (32.3%)	-0.065 (-0.100, -0.029)	0.997 (0.861, 1.156)	0.970
Secondary Outcomes						
**Cerebral Infarction**	6 months**	–	–	–	–	–
	1 year	19/1,091 (1.7%)	14/1,109 (1.3%)	0.005 (-0.005, 0.015)	1.674 (0.838, 3.343)	0.140
	2 years	23/1,091 (2.1%)	24/1,109 (2.2%)	-0.001 (-0.013, 0.012)	1.395 (0.783, 2.486)	0.257

**TIA**	6 months**	–	–	–	–	–
	1 year**	–	–	–	–	–
	2 years	12/1,139 (1.1%)	23/1,180 (1.9%)	-0.009 (-0.019, 0.001)	0.740 (0.366, 1.493)	0.399
**MI**	6 months**	–	–	–	–	–
	1 year	20/1,048 (1.9%)	39/1,074 (3.6%)	-0.017 (-0.031, -0.003)	0.614 (0.358, 1.054)	0.074
	2 years	29/1,048 (2.8%)	52/1,074 (4.8%)	-0.021 (-0.037, -0.005)	0.756 (0.479, 1.194)	0.229
**Composite *MACE**	6 months	27/924 (2.8%)	48/957 (5.0%)	-0.021 (-0.039, -0.003)	0.619 (0.387, 0.993)	0.044
	1 year	48/924 (5.2%)	84/957 (8.8%)	-0.036 (-0.059, -0.013)	0.692 (0.485, 0.987)	0.041
	2 years	67/924 (7.3%)	128/957 (13.4%)	-0.061 (-0.089, -0.034)	0.751 (0.558, 1.012)	0.059
**Cardioversion**	6 months	13/976 (1.3%)	27/1,053 (2.6%)	-0.012 (-0.024, -0.000)	0.542 (0.280, 1.051)	0.066
	1 year	14/976 (1.4%)	37/1,053 (3.5%)	-0.021 (-0.034, -0.007)	0.440 (0.238, 0.814)	0.007
	2 years	22/976 (2.3%)	46/1,053 (4.4%)	-0.021 (-0.037, -0.006)	0.622 (0.373, 1.036)	0.066
**AF Ablation**	6 months**	–	–	–	–	–
	1 year	13/1,088 (1.2%)	30/1,159 (2.6%)	-0.014 (-0.025, -0.003)	0.537 (0.280, 1.031)	0.352
	2 years	26/1,088 (2.4%)	40/1,159 (3.5%)	-0.011 (-0.025, 0.003)	0.929 (0.565, 1.526)	0.77
**AKI**	6 months	29/844 (3.4%)	49/869 (5.6%)	-0.022 (-0.042, -0.002)	0.648 (0.409, 1.026)	0.062
	1 year	45/844 (5.3%)	76/869 (8.7%)	-0.034 (-0.058, -0.010)	0.698 (0.483, 1.010)	0.055

	2 years	59/844 (7.0%)	110/869 (12.7%)	-0.057 (-0.085, -0.029)	0.715 (0.520, 0.983)	0.038

Abbreviations: OACs = oral anticoagulants; RD = risk difference; HR= Hazard Ratio; CI = confidence interval; MACE = major adverse cardiovascular events.

** MACE* is a composite outcome including **mortality, ischemic stroke, and acute myocardial infarction**.

AF, atrial fibrillation; AKI, acute kidney injury; ED, emergency department; MACE,MI, myocardial infarction; TIA, transient ischemic attack.

HFpEF = Heart Failure with preserved Ejection Fraction

** Counts suppressed by TriNetX
